# Attitudes Toward Digital Meal Assistance Services Among Older Adults in China: Cross-Sectional Survey

**DOI:** 10.2196/84956

**Published:** 2026-03-30

**Authors:** Zhe Chen, Tinghe Deng, Xiaoli Zhu

**Affiliations:** 1 Department of Economics and Management, Century College, Beijing University of Posts and Telecommunications, Beijing, China; 2 Chinese Center for Health Education, Building 12, Anhua Xili First District, Chaoyang District, Beijing, 100011, China, 86 10 64266955; 3 Overseas Chinese College, Capital University of Economics and Business, Beijing, China

**Keywords:** digital meal assistance services, older adults, behavioral attitudes, usage intention, actual use, China

## Abstract

**Background:**

Digital eldercare services are promoted to address gaps in traditional care provision, yet actual uptake among older adults remains limited. Prior research has largely focused on technological attributes, with less attention to older adults’ context-specific behavioral attitudes toward digital services.

**Objective:**

This study aimed to identify the multidimensional structure of older adults’ behavioral attitudes toward digital meal assistance services and to examine their associations with usage intention and actual use.

**Methods:**

A mixed-mode survey was conducted in 2020 among adults aged 60 years or older in a large metropolitan city in China. A total of 1019 valid questionnaires were collected (n=405 online and n=614 offline). Exploratory factor analysis was used to identify attitudinal dimensions, followed by binary logistic regression analyses conducted separately for online and offline samples, controlling for sociodemographic characteristics.

**Results:**

Five attitudinal dimensions were identified, explaining 60.38% of the variance. Regression analyses showed that associations between attitudinal dimensions and service use differed by usage stage and sample type. Digital trust was the most consistent predictor, significantly associated with intention to use in both samples—particularly online (odds ratio [OR] 1.351, 95% CI 1.054‐1.732; *P*=.02)—and with actual use in the offline sample (OR 1.068, 95% CI 1.011‐1.128; *P*=.02), with a marginal association online (OR 1.347, 95% CI 0.986‐1.840; *P*=.06).

**Conclusions:**

Older adults’ adoption of digital meal assistance services is shaped by multidimensional and context-dependent attitudes that extend beyond technological considerations. Policy efforts should therefore emphasize trust-building, affordability, and differentiated strategies tailored to diverse groups of older adults.

## Introduction

Digital older care services are increasingly viewed as an important approach to addressing population aging and alleviating shortages in traditional eldercare provision. Prior studies indicate that digital technologies, such as the internet and big data, can integrate service resources through smart platforms. These platforms enable the delivery of diverse services tailored to older adults’ needs, including home-based care, remote medical consultation, rehabilitation guidance, and health management [1,2]. In China, national policies have further promoted the integration of digital technologies and eldercare services. The Smart Health and Elderly Care Industry Development Action Plan (2021‐2025) explicitly encourages the digitalization and age-friendly application of services such as meal delivery and household assistance, aiming to narrow the digital divide among older adults [3]. Despite these policy efforts and technological advances, the actual use of digital eldercare services among older adults remains limited [2,4].

Digital meal assistance services represent a key component of digital eldercare by linking daily nutritional support with the broader eldercare service delivery system through platform-based models. Meals on Wheels services were initially designed for older adults with difficulties in meal preparation and have gradually expanded to serve broader populations [5,6]. Existing research suggests that meal assistance services can improve nutritional intake and health outcomes, reduce family caregiving burden, and promote social interaction [7-9]. With digitalization, these services have evolved into multiple forms, including online meal delivery platforms and food delivery applications [10-12]. However, empirical evidence indicates that such services have not been widely adopted by older adults, potentially undermining food security and health outcomes [2,7,10].

Previous studies have mainly relied on the Andersen behavioral model, the Technology Acceptance Model (TAM), and the theory of planned behavior (TPB) to explain digital service use, highlighting the roles of perceived usefulness, perceived ease of use, and behavioral attitudes in shaping usage intention [13-16]. Nevertheless, 2 gaps remain. First, many studies focus on the general population and give insufficient attention to older adults, who differ markedly in digital skills and risk perceptions [10,17]. Second, although behavioral attitudes are frequently emphasized, their underlying structure among older adults remains insufficiently specified, and the mechanisms through which attitudes translate into actual service use—the intention-behavior gap—are not fully understood [14,16,18].

To address these gaps, this study examines digital meal assistance services in urban China using survey data collected from older adults in 2020. We aim to (1) identify the structure of older adults’ behavioral attitudes toward digital meal assistance services through exploratory factor analysis (EFA); (2) examine differences in attitudinal structures across population subgroups; and (3) assess the associations between different attitudinal dimensions, usage intention, and actual service use. By adopting a behavioral attitude perspective, this study seeks to clarify the mechanisms underlying digital eldercare service adoption among older adults and to provide empirical insights for service design and policy development.

## Methods

### Study Design and Setting

This study used data from a questionnaire survey examining older adults’ intention to use smart eldercare services. The survey was conducted over a 2-month period in 2020 in 2 districts of a large metropolitan city in China. The survey collected information on respondents’ sociodemographic characteristics and their awareness and use of digital meal assistance services (ie, online food ordering services). Given potential systematic differences between online and offline surveys in digital skills and service access, a mixed-mode design was adopted. Descriptive and regression analyses were conducted separately for online and offline samples. The final analytic sample comprised 1019 valid responses (n=405 online and n=614 offline). Additional details on the sampling framework and survey implementation are provided in Multimedia Appendix 1.

### Offline Survey Procedures

The offline survey used a 2-stage stratified random sampling strategy. Streets within each district were first selected using simple random sampling. Communities within selected streets were then stratified by geographic accessibility to community eldercare service stations (approximately 15-min walkability, defined as about 1.0‐1.2 km based on navigation distance) and randomly sampled within each stratum. Eligible older adults were recruited through intercept surveys, yielding 614 valid questionnaires. Further procedural details are provided in Multimedia Appendix 1.

### Online Survey Procedures and Quality Control

The online survey was administered via the Wenjuanxing platform and yielded 405 valid responses. Survey links were primarily distributed by community staff, with additional responses collected through the platform’s mutual-assistance service. No incentives were provided. Eligibility was ensured through an automated age-screening item (≥60 y), and duplicate submissions were prevented by restricting 1 response per IP address. Participation was voluntary, and informed consent was implied by questionnaire submission. Additional quality control details are provided in Multimedia Appendix 1, and the completed CHERRIES (Checklist for Reporting Results of Internet E-Surveys) checklist is provided in Checklist 1.

### Measures

#### Behavioral Attitudes Toward Digital Meal Services

According to the TPB, behavioral attitudes are derived from individuals’ beliefs about behavioral outcomes and are influenced by perceived behavioral control [19]. When individuals perceive that they have sufficient ability and resources to perform a behavior, they are more likely to develop positive attitudes. The TAM further posits that perceived usefulness and perceived ease of use are core mechanisms underlying attitude formation, with external factors influencing attitudes indirectly through these perceptions [20-22]. Building on the TPB and the TAM, this study incorporates the digital divide experienced by older adults as well as the family-oriented eldercare context in China [23-25]. Behavioral attitudes are defined as older adults’ overall evaluations of the value, acceptability, and safety of digital meal assistance services within a specific service context, as well as their perceived impacts on themselves and their families. From this perspective, attitudes toward digital meal assistance services encompass evaluations of usefulness and convenience. They also involve judgments related to family roles and caregiving responsibilities, trust-related risk assessments, and perceived constraints associated with resources and affordability.

Measurement items were developed based on a systematic review of prior studies on eldercare service use, digital technology adoption, and digital inequality. The initial item pool primarily covered perceived service value, service acceptance, safety concerns, and perceived impacts on individuals and families. To reduce social desirability and self-presentation bias, some items were framed in a projective manner (eg, asking how “older adults around you” perceive digital meal services). Reverse-coded items were also included to minimize acquiescence bias. All items were measured using a 5-point Likert scale ranging from 1 (“strongly disagree”) to 5 (“strongly agree”). Following semantic refinement and standardization prior to the formal survey, a total of 19 items were retained for analysis.

Based on the results of EFA, composite indices were constructed for each behavioral attitude dimension. Scores for each dimension were calculated as the mean of the corresponding items, with higher scores indicating more positive attitudes. Internal consistency for each attitudinal dimension was assessed using Cronbach α.

#### Outcome Variables

To assess the effects of behavioral attitudes, 2 outcome variables were examined: intention to use digital meal assistance services and actual service use. Usage intention was measured using the item “Would you (continue to) order meals through internet-based platforms?” on a 5-point Likert scale (1=“definitely would not use,” 5=“definitely would use”) and dichotomized as 1 for responses of 4 or 5 and 0 otherwise. Actual service use was measured by the frequency of online food ordering and coded as 1 for any reported use and 0 for “never.”

### Ethical Considerations

This study involved an anonymous questionnaire survey administered online (via Wenjuanxing) and offline face-to-face. No personally identifiable information (eg, names, contact details, identification numbers, or precise addresses) or sensitive personal data (eg, medical or financial information) was collected, and no intervention, clinical procedures, or biological sample collection was involved. Under Article 32 of the Measures for Ethical Review of Life Sciences and Medical Research Involving Human Subjects, research using anonymized information data that does not cause harm to participants and does not involve sensitive personal information or commercial interests may be exempt from ethics review. Accordingly, this anonymous, minimal-risk survey study met the criteria for exemption from institutional ethics review.

Participants were informed about the study purpose, procedures, voluntary participation, and data protection. For the online survey, consent was implied by submission of the completed questionnaire; for the offline survey, oral informed consent was obtained prior to participation. Participants could withdraw at any time without consequences. No compensation was provided for participation.

### Data Privacy and Security

All survey data were collected anonymously. Online responses were exported and stored in password-protected files, and offline paper questionnaires (if applicable) were securely stored and then digitized. Only the research team had access to the data.

### Data Analysis

EFA was conducted to identify the latent structure of older adults’ behavioral attitudes toward digital meal assistance services. Prior to analysis, questionnaires with missing values on key variables or substantial nonresponse were excluded. The final analytic sample consisted of 1019 complete questionnaires; therefore, no imputation was applied.

EFA followed standard procedures of item screening, factor extraction, rotation, and interpretation. Factors were extracted using principal component analysis, with the number of factors determined by the Kaiser criterion (eigenvalues >1). Varimax orthogonal rotation was applied. Items with factor loadings below 0.50 were considered for removal; items with cross-loadings (loading differences <0.20 across factors) or communalities below 0.40 were also evaluated and excluded when appropriate.

Binary logistic regression models were used to examine associations between behavioral attitude dimensions and both usage intention and actual service use. Given differences between online and offline samples, all models were estimated separately for each subsample. The general model specification was


(1) $$log\left(\frac{p_{i}}{1-p_{i}}\right)=\beta_{0}+\beta_{1}X_{i}+\beta_{2}Z_{i}$$

where $p_{i}$ denotes the probability that individual i reports a higher usage intention or actual service use; $X_{i}$ represents behavioral attitudes (or specific attitudinal dimensions); and $Z_{i}$ includes control variables (gender, age, education, and income). Results were reported as odds ratios (ORs) with 95% CIs. Given the sensitivity of the Hosmer-Lemeshow test in large samples, pseudo*-R*² and the Akaike information criterion were also reported as supplementary fit indicators.

## Results

### Participant Characteristics

Table 1 summarizes the sociodemographic characteristics of the sample. Overall, the age distribution was skewed toward younger older adults, with participants aged 60‐69 years accounting for 569 of 1019 (55.8%) of the sample, which may limit generalizability. Compared with the offline sample, the online sample was younger on average, whereas the offline sample included a higher proportion of the oldest-old (aged ≥80 y). In terms of socioeconomic characteristics, the online sample had higher educational attainment than the offline sample, with 272 of 405 (67.2%) reporting a high school education or above compared with 297 of 614 (48.4%) in the offline sample. The online sample also had a higher proportion of high-income respondents (≥8000 CNY, 1 CNY=US $0.14), whereas the offline sample included a relatively larger share of middle-income respondents (4000‐7999 CNY). Overall, the offline sample more closely resembled community-dwelling older adults, whereas the online sample was more likely to represent younger and more digitally skilled individuals. Accordingly, the findings were more applicable to younger and older adults and those with greater digital capacity, and caution was warranted when generalizing to the oldest-old or to older adults with limited digital skills; these limitations are discussed in the Limitations section.

**Table 1.** Sample characteristics of the study participants (n=1019).

Variables and categories	Total sample, n (%)^a^	Online survey, n (%)^a^	Offline survey, n (%)^a^
Gender			
Male	477 (46.815)	223 (55.062)	254 (41.368)
Female	542 (53.185)	182 (44.938)	360 (58.632)
Education level			
No formal education	97 (9.519)	3 (0.741)	94 (15.309)
Primary school	82 (8.048)	8 (1.975)	74 (12.052)
Junior high school	271 (26.595)	122 (30.123)	149 (24.266)
Senior high school or above	569 (55.838)	272 (67.160)	297 (48.373)
Age group (y)			
60‐69	569 (55.838)	212 (52.346)	357 (58.143)
70‐79	365 (35.819)	173 (42.716)	192 (31.270)
≥80	85 (8.342)	20 (4.938)	65 (10.587)
Self-rated health			
Very poor	106 (10.402)	38 (9.383)	68 (11.074)
Poor	40 (3.926)	24 (5.926)	16 (2.606)
Fair	139 (13.640)	91 (22.469)	48 (7.820)
Good	538 (52.796)	153 (37.778)	385 (62.703)
Very good	196 (19.235)	99 (24.444)	97 (15.798)
Marital status			
Married	850 (83.415)	361 (89.136)	489 (79.641)
Unmarried	63 (6.183)	23 (5.679)	40 (6.514)
Widowed	106 (10.402)	21 (5.185)	85 (13.844)
Monthly income (CNY^b^)			
0‐3999	213 (20.902)	92 (22.716)	121 (19.706)
4000‐7999	485 (47.596)	124 (30.617)	361 (58.795)
≥8000	321 (31.502)	189 (46.667)	132 (21.498)

a Percentages may not sum to 100% due to rounding.

b CNY: Chinese yuan; 1 CNY=US $0.14.

### Behavioral Attitude Structure

EFA was conducted to identify the latent structure of older adults’ behavioral attitudes toward digital meal assistance services. Data suitability was confirmed by a significant Bartlett test of sphericity (*χ*²=286.67, *P*<.01) and a Kaiser-Meyer-Olkin value of 0.894, indicating adequate interitem correlations. After excluding 3 items based on predefined criteria, 16 items were retained for analysis. Five factors with eigenvalues greater than 1 were extracted, with initial eigenvalues of 6.01, 2.43, 1.82, 1.39, and 1.03. Following Varimax orthogonal rotation, the 5 factors explained 14.60%, 14.13%, 11.64%, 11.09%, and 8.92% of the total variance, respectively, yielding a cumulative explained variance of 60.38%. Detailed results are presented in Table 2.

Table 3 presents the standardized factor loadings and communalities for each retained item. All items showed strong loadings on their corresponding factors, and each factor was represented by at least 3 items, indicating a clear and stable factor structure. Reliability analysis showed that the overall scale demonstrated good internal consistency, with a Cronbach α of 0.879. In addition, correlations between the mean scores of individual behavioral attitude dimensions and the overall scale mean ranged from 0.414 to 0.762, suggesting that the dimensions were moderately correlated while maintaining adequate discriminant validity (Multimedia Appendix 2).

**Table 2.** Rotated total variance explained.^a^

Factor	Eigenvalue (rotated)	Variance explained (%)	Cumulative variance explained (%)
F1	3.065	14.60	14.60
F2	2.968	14.13	28.73
F3	2.444	11.64	40.37
F4	2.330	11.09	51.46
F5	1.873	8.92	60.38

a Variance explained is based on principal component analysis with Varimax rotation.

**Table 3.** Exploratory factor analysis loading matrix.^a^

Item	F1	F2	F3	F4	F5	Communality
I am likely to use online meal ordering services when cooking on my own is difficult	0.773	—^b^	—	—	—	0.754
I am likely to use online meal ordering services when conditions for cooking at home are unfavorable or when I do not wish to cook	0.679	—	—	—	—	0.627
I am likely to use online meal ordering services when my children experience difficulty in providing me with meals	0.649	—	—	—	—	0.529
I am likely to use online meal ordering services when going out is inconvenient due to extreme weather	—	0.717	—	—	—	0.726
I am likely to use online meal ordering services for convenience and to save time and effort	—	0.718	—	—	—	0.664
I am likely to use online meal ordering services when the food I want is complicated to prepare	—	0.686	—	—	—	0.562
I avoid using online meal ordering services because of concerns about being perceived as lazy by others	—	—	0.769	—	—	0.779
I avoid using online meal ordering services because of concerns that it may reduce children’s sense of responsibility	—	—	0.724	—	—	0.701
I avoid using online meal ordering services due to concerns about upsetting children	—	—	0.723	—	—	0.678
I avoid using online meal ordering services because I am not accustomed to ordering food online	—	—	—	0.608	—	0.612
I am more likely to use online meal ordering services when people of my age around me use such services	—	—	—	0.713	—	0.725
I avoid using online meal ordering services because I am concerned about making operational mistakes or being scammed	—	—	—	0.704	—	0.711
I avoid using online meal ordering services because of concerns about food quality or hygiene during preparation	—	—	—	0.691	—	0.606
I avoid using online meal ordering services if my children have to pay for the cost	—	—	—	—	0.652	0.662
I am more likely to use online meal ordering services when government subsidies are available	—	—	—	—	0.583	0.584
I am willing to use online meal ordering services only when my pension income is sufficient to cover the cost	—	—	—	—	0.671	0.681

a Only factor loadings with absolute values ≥0.50 are reported. Factor extraction was based on principal component analysis with Varimax rotation.

b Not reported (factor <0.50).

Based on the content characteristics of items with high factor loadings, 5 factors were labeled. Alleviating burden (F1) reflects value-based judgments regarding whether digital meal assistance services were worth using, particularly in terms of their potential to reduce the caregiving burden on adult children in daily life. When such services were perceived as helping to alleviate family caregiving pressure, older adults tended to hold more positive attitudes toward their use. Enhancing convenience (F2) captures perceived evaluations of the extent to which digital meal assistance services improve convenience in older adults’ daily lives. This factor reflected the functional use of the service, consistent with perceived usefulness in the TAM. Service acceptance (F3) represented older adults’ evaluations of the social meaning of digital meal assistance services. When service use was interpreted as indicating that adult children had not adequately fulfilled their caregiving responsibilities, attitudes toward the service tended to be negative. Digital trust (F4) reflected older adults’ attitudinal judgments regarding the safety and reliability of digital meal assistance services. Funding sources (F5) captured older adults’ perceived affordability of digital meal assistance services, consistent with perceived behavioral control in the TPB. Lower perceived affordability was associated with a lower likelihood of service use.

Notably, perceived ease of use, as defined in the TAM, did not emerge as an independent attitudinal dimension in the identified factor structure.

### Differences in Behavioral Attitudes Across Groups

Table 4 summarizes mean scores across behavioral attitude dimensions by participant characteristics. Overall, older adults reported relatively higher evaluations on alleviating burden (F1) and enhancing convenience (F2). Middle- and higher-income groups generally scored higher on enhancing convenience (F2), service acceptance (F3), and digital trust (F4), whereas lower-income older adults reported higher scores on funding sources (F5) and alleviating burden (F1). Higher educational attainment was associated with more positive evaluations of alleviating burden (F1), enhancing convenience (F2), and service acceptance (F3), as well as lower concern regarding funding sources (F5). By contrast, differences in digital trust (F4) were modest across education groups. By survey mode, the online sample consistently reported higher scores across most attitude dimensions—particularly enhancing convenience (F2)—whereas differences in digital trust (F4) between online and offline samples were relatively small.

**Table 4.** Mean scores of behavioral attitude dimensions across different groups of older adults.^a^

Behavioral attitude dimension	Total sample	Middle- or high-income^b^	Low-income^b^	Higher education	Lower education^c^	Offline sample^d^	Online sample^d^
Alleviating burden (F1), mean (SD)	3.668 (1.412)	3.278 (1.357)	3.816 (1.602)	3.700 (1.288)	3.346 (1.492)	3.278 (1.525)	3.417 (1.064)
Enhancing convenience (F2), mean (SD)	3.588 (1.500)	3.763 (1.410)	3.237 (1.762)	3.604 (1.287)	3.251 (1.650)	3.054 (1.682)	3.611 (0.784)
Service acceptance (F3), mean (SD)	3.334 (1.890)	3.411 (1.999)	3.146 (1.35)	3.551 (2.029)	3.228 (1.681)	3.209 (2.00)	3.441 (0.784)
Digital trust (F4), mean (SD)	3.022 (1.226)	3.082 (1.023)	2.927 (1.79)	2.962 (1.228)	3.002 (1.219)	2.989 (1.32)	3.132 (1.063)
Funding sources (F5), mean (SD)	3.010 (1.947)	2.711 (1.953)	3.132 (1.812)	3.002 (2.140)	3.359 (1.703)	2.796 (1.961)	3.364 (1.807)

a Values represent mean scores on a 5-point Likert scale (1=strongly disagree, 5=strongly agree).

b Low-income refers to a monthly income of 0-3999 CNY; middle- or high-income refers to ≥4000 CNY; 1 CNY=US $0.14.

c Lower education refers to junior high school or below; higher education refers to high school or above.

d Offline and online samples were collected using different survey modes as described in the Methods section.

### Associations Between Behavioral Attitudes, Usage Intention, and Actual Use

Tables5  6 present logistic regression results examining associations between the 5 behavioral attitude dimensions and both intention to use and actual use of digital meal assistance services, estimated separately for the online and offline samples. All 4 models were statistically significant (*P*<.001), with pseudo*-R*² values ranging from 0.156 to 0.240 and Akaike information criterion values from 800.001 to 1002.470, indicating acceptable model fit. Hosmer-Lemeshow tests did not suggest a significant lack of fit (*P* value range .055-.062).

**Table 5.** Logistic regression results for the associations between behavioral attitude dimensions, intention to use, and actual use of digital meal assistance services.

Variables	Panel A, online sample (n=405)		Panel B, offline sample (n=614)	
	Intention to use	Actual use	Intention to use	Actual use
Alleviating burden (F1), OR^a^ (95% CI; *P* value)	1.618^b^ (1.235-2.118; <.001)	1.742^b^ (1.286-2.359;<.001)	1.673^b^ (1.420-1.969;<.001)	2.340 (0.710-7.727; .16)
Enhancing convenience (F2), OR (95% CI; *P* value)	1.107^c^ (1.002-1.223; .046)	1.089 (0.900-1.318; .39)	1.129 (0.968-1.317; .13)	0.843 (0.652-1.088; .19)
Service acceptance (F3), OR (95% CI; *P* value)	1.128 (0.979-1.299; .10)	1.250^c^ (1.000-1.557; .05)	1.123^c^ (1.001-1.259; .049)	1.011 (0.991-1.031; .27)
Digital trust (F4), OR (95% CI; *P* value)	1.351^c^ (1.054-1.732; .02)	1.347^d^ (0.986-1.840; .06)	1.221^b^ (1.083-1.376; <.001)	1.068 (1.011-1.128; .02)
Funding sources (F5), OR (95% CI; *P* value)	1.417^d^ (0.957-2.100; .08)	0.800 (0.444-1.440; .46)	0.806^d^ (0.642-1.020; .07)	0.600 (0.225-1.598; .31)
Prior use of online meal ordering, OR (95% CI; *P* value)	6.350^b^ (3.668‐10.973;<.001)	N/A^e^	4.773^b^ (3.523-6.471;<.001)	N/A
Log likelihood ratio	202.94	67.1	354.25	285.85
Model significance (*P* value)	<.001^b^	<.001^b^	<.001^b^	<.001^b^
Pseudo R²	0.212	0.156	0.24	0.228
AIC^f^	800.001	888.224	1002.47	979.838
Hosmer-Lemeshow goodness-of-fit (*P* value)^g^	.06	.06	.06	.06

a OR: odds ratio.

b *P*≤.001*.*

c *P*≤.05*.*

d *P*<.10.

e NA: not applicable.

f AIC: Akaike information criterion.

g The Hosmer-Lemeshow goodness-of-fit test is sensitive to large sample sizes; therefore, pseudo *R*² and Akaike information criterion are reported as complementary indicators of model fit.

**Table 6.** Control variables included in all models (definitions, distinctions, and coding).

Control variables^a^	What it means (to distinguish similarly named variables)	Coding/categories^b^
Gender	Binary indicator	Male=1; female=0
Education level	Highest attained education category (not y of schooling)	No formal education=1; primary school=2; junior high school=3; high school and above=4
Age group	Categorical age bands (not continuous age in y)	60-69=1; 70-79=2; ≥80=3
Self-rated health	5-point subjective health rating	Very poor=1 to very good=5
Marital status	Current marital status	Married=1; unmarried/widowed=0
Monthly household income (CNY)^c^	Household-level total monthly income (not personal income)	0-3999 CNY=1; 4000-7999 CNY=2; ≥8000 CNY=3

a Control variables were included in all models reported in Table 5.

b For binary variables, the reference category is coded as 0 (female; unmarried/widowed). For ordinal variables entered as numeric scores (education level, age group, self-rated health, and monthly household income), odds ratios represent the change in odds per 1-level increase in the coded category.

c Exchange rate for interpretation (study period Jul-Aug 2020): 1 CNY=US $0.14.

In the online sample, alleviating burden (F 1) was positively associated with both usage intention and actual use. A 1-unit increase in F1 was associated with a 61.8% increase in the odds of intending to use the service (OR 1.618, 95% CI 1.235‐2.118; *P*<.001) and a 74.2% increase in the odds of actual use (OR 1.742, 95% CI 1.286‐2.359; *P*<.001). In addition, digital trust (F4) was positively associated with usage intention, with each 1-unit increase corresponding to a 35.1% increase in the odds of intention to use (OR 1.351, 95% CI 1.054‐1.732; *P*=.02), and showed a marginal association with actual use (OR 1.347, 95% CI 0.986‐1.840; *P*=.06). Service acceptance (F3) was not significantly associated with usage intention (*P*=.10) but demonstrated a marginal positive association with actual use (OR 1.250, 95% CI 1.000‐1.557; *P*=.05).

In the offline sample, alleviating burden (F1), service acceptance (F3), and digital trust (F4) were all positively associated with usage intention. Notably, a 1-unit increase in digital trust (F4) was associated with a 22.1% increase in the odds of intention to use (OR 1.221, 95% CI 1.083‐1.376; *P*<.001). In contrast, funding sources (F5) showed a marginal negative association with usage intention (OR 0.806, 95% CI 0.642‐1.020; *P*=.07). With respect to actual use, digital trust (F4) was the only attitude dimension that remained significantly associated with service use in the offline sample, with a 1-unit increase corresponding to a 6.8% increase in the odds of actual use (OR 1.068, 95% CI 1.011‐1.128; *P*=.02). Although the odds ratio for alleviating burden (F1) in predicting actual use appeared large in the offline sample, this association did not reach statistical significance, likely reflecting limited statistical power and greater heterogeneity in offline respondents.

## Discussion

This study uses digital meal assistance services as a case to explain the phenomenon of “adequate supply but insufficient use” of digital eldercare services. Based on survey data from 1019 older adults in a large metropolitan city in China, we examined service adoption from a behavioral attitude perspective. The results show that older adults’ attitudes toward digital meal assistance services have a multidimensional structure. Different attitudinal dimensions play distinct roles in shaping usage intention and actual service use. Evaluations of digital eldercare services are not determined by technology alone. They are also influenced by family caregiving contexts, perceived social norms, risk perceptions, and financial constraints. In addition, the effects of behavioral attitudes differ across the transition from intention to actual behavior. These findings provide empirical evidence to inform the promotion of digital services in an aging society.

Older adults’ behavioral attitudes toward digital meal assistance services exhibited a clear multidimensional structure. Five dimensions were identified: Alleviating burden (F1), enhancing convenience (F2), service acceptance (F3), digital trust (F4), and funding sources (F5). These findings indicate that evaluations of digital eldercare services extend beyond functional attributes. Family caregiving relationships, perceived social norms, risk perceptions, and financial constraints also shape these evaluations. This pattern is consistent with prior studies showing that older adults’ technology adoption is influenced by broader social and contextual factors [26-28]. A purely technology-rational framework that emphasizes usefulness or ease of use alone may therefore be insufficient to explain service choice. Notably, no independent factor of perceived ease of use emerged in this study. Ease of use appears to be embedded within broader evaluations of convenience, trust, and affordability. This suggests that, in specific eldercare service contexts, older adults’ attitudinal structures toward technology may differ from those proposed in classical TAMs.

Attitudinal priorities toward digital meal assistance services varied across older adult groups. Most older adults focused on whether these services could help alleviate caregiving burdens, whereas younger-old adults placed greater emphasis on support for daily convenience. As shown in Table 4, the offline sample scored higher on alleviating burden (F1) than on other dimensions, indicating that many older adults tend to view digital meal assistance services as a supplement to the family caregiving system. This pattern is consistent with the Chinese eldercare context, in which limited family caregiving resources, particularly from spouses and adult children, have been an important foundation for the development of formal eldercare services [29,30]. Accordingly, the perceived ability of digital meal assistance services to reduce family caregiving pressure serves as a key reference in older adults’ attitude formation. By contrast, the online sample scored higher on enhancing convenience (F2), consistent with previous studies [31-33]. This finding indicates that younger old adults place greater value on digital eldercare services that improve daily convenience and support autonomy. It also suggests that future cohorts of older adults may increasingly emphasize the role of digital services in enhancing quality of life and independent living.

Insufficient digital trust represents a key barrier to the use of digital meal assistance services, particularly among lower-income older adults. The findings show that although low-income older adults expressed relatively high levels of service acceptance (F3), their scores on digital trust (F4) were lower than those of other income groups. This attitudinal pattern is consistent with the regression results, which showed that digital trust was significantly associated with usage intention and selected aspects of actual service use. These findings align with previous research indicating that the use of eldercare services tends to be concentrated among middle- and high-income groups [34-36]. They also shed light on the mechanism through which the digital divide operates. Limited digital skills and resources not only constrain older adults’ capacity to use digital services but may also weaken trust, thereby influencing usage decisions. When individuals face simultaneous deficits in digital skills and resources, psychological strain may increase, including reduced trust, which can further limit service use and exacerbate digital inequality [37,38]. Notably, regardless of income level, overall scores on digital trust remained relatively low among older adults, consistent with the distribution shown in Table 4. This pattern highlights trust as a fundamental condition that requires particular attention in the broader promotion of digital eldercare services.

Behavioral attitudes played an important but uneven role in the translation of usage intention into actual behavior. Although the TPB posits behavioral intention as a key predictor of behavior, prior studies have reported inconsistencies in how attitudes relate to intention and actual use [39,40]. In the online sample, alleviating burden (F1) was significantly associated with both usage intention and actual service use. Service acceptance (F3) was associated only with actual use and showed no significant relationship with usage intention. This pattern suggests that some attitudinal dimensions may be less salient during intention formation but exert a more direct influence on usage decisions. By contrast, in the offline sample, multiple attitudinal dimensions, including alleviating burden (F1), service acceptance (F3), and digital trust (F4), were significantly associated with the usage intention, whereas only digital trust was further translated into actual service use. Funding sources (F5) primarily influenced usage intention and showed no significant association with actual behavior. Overall, behavioral attitudes toward digital eldercare services show clear context dependence across intention and behavior. This provides empirical evidence for the intention–behavior gap in older adults’ use of digital services.

These findings have important implications for policy and practice. Greater attention should be given to the vulnerability of low-income older adults in digital environments. Public digital spaces and smart communities can improve access to digital technologies for groups affected by the digital divide [41,42]. Community-based courses and remote assistance programs can support tailored digital skills training [43]. Digital platforms should also reduce barriers through age-friendly interface design, such as larger fonts and simplified procedures [41,44]. In addition, efforts are needed to reduce technology-related anxiety among older adults [45-47]. Trust-enhancing mechanisms, including service quality certification, data security standards, and transparent pricing, can strengthen perceived safety and confidence. Finally, digital eldercare services should better match the needs of different groups. Services should emphasize burden reduction for most older adults and highlight independence and convenience for younger-old or higher-income groups. For low-income older adults, establishing basic trust remains a critical condition for service uptake.

This study has several limitations. First, the sample was skewed toward younger older adults, with relatively few participants aged 80 years or older. The findings may therefore be less representative of the oldest-old population. Second, differences between the online and offline samples may have introduced selection bias. Although key control variables were included, the results reflect associations rather than causal relationships. In addition, the sample was drawn from a single region, which may limit generalizability. Future studies should include larger and more diverse samples, increase representation of the oldest-old, and apply methods such as sample weighting or sensitivity analyses to better assess potential sampling bias.

## Supplementary material

10.2196/84956 Multimedia Appendix 1 Sampling and fieldwork procedures.

10.2196/84956 Multimedia Appendix 2 Correlations between behavioral attitude dimensions and the overall scale score.

10.2196/84956 Checklist 1 CHERRIES checklist.

## Abbreviations

CHERRIES: Checklist for Reporting Results of Internet E-Surveys
OR: odds ratio
TAM: Technology Acceptance Model
