# Supplementary material for: Attitudes Toward Digital Meal Assistance Services Among Older Adults in China: Cross-Sectional Survey
Source: JMIR Aging. 2026 Mar 30;9:e84956. doi: 10.2196/84956 (PMC13035262; doi:10.2196/84956)
Supplement: Multimedia Appendix 1 [file aging-v9-e84956-s001.pdf]

Multimedia Appendix 1

Multimedia Appendix 1. Sampling and fieldwork procedures

To cover older adults with different levels of digital capability and varying channels of service exposure, and to account for potential systematic differences between online and offline samples, we conducted a mixed-mode questionnaire survey (online and offline). Analyses were conducted separately for online and offline samples.

A1. Online survey (Wenjuanxing)

Survey platform: Wenjuanxing (a third-party online survey platform).

Target population: Adults aged ≥60 years in City S.

Distribution and responses: A total of 420 questionnaires were distributed, and 405 valid responses were retained after quality checks.

Quality control: Eligibility screening items were implemented for age and residence. Respondents younger than 60 years were automatically exited from the questionnaire. The platform restricted submissions to one response per IP address to prevent multiple entries. Invalid responses were excluded based on completion time and response consistency checks.

A2. Offline survey: stratified two-stage random sampling

The offline survey was conducted in two stages: street selection and community selection.

Stage 1: Street selection (sampling unit: streets)

1. We compiled a complete list of streets in each study district.
2. Streets were assigned numeric codes.
3. The coded street list was entered into a computer program, and a simple random sample of streets was selected for the second stage.

Stage 2: Community selection (sampling unit: communities)

Within selected streets, communities were sampled using geographic stratification based on walkability to a community elderly care service station.

Defining the center point: The community elderly care service station within each street was used as the center point. If multiple stations existed, the main station with the largest coverage (or the most centrally located station) was selected.

Geographic stratification: Communities were stratified into two layers based on walkability to the center point: (1) non-peripheral areas (reachable within a 15-minute walk) and (2) peripheral areas (not reachable within a 15-minute walk). Walkability was determined based on map navigation distance, approximately 1.0–1.2 km.

Random selection within strata: Communities within each stratum were coded, and a random sample of communities was selected within each stratum using computer-based randomization.

A3. Intercept survey within selected communities

Field investigators approached eligible older adults (aged ≥60 years) in public areas within the selected communities (eg, community squares, activity rooms, entrances/exits). After obtaining informed consent, respondents completed the questionnaire through face-to-face administration.

To reduce potential selection bias associated with intercept sampling, interviewers received standardized training and followed a predefined fieldwork protocol. Survey times and locations were dispersed to avoid over-recruitment from specific settings or subgroups.

A4. Final sample size

Valid online sample: 405

Valid offline sample: 614

Total valid sample: 1,019
