# Supplementary material for: Attitudes Toward Digital Meal Assistance Services Among Older Adults in China: Cross-Sectional Survey
Source: JMIR Aging. 2026 Mar 30;9:e84956. doi: 10.2196/84956 (PMC13035262; doi:10.2196/84956)
Supplement: Multimedia Appendix 2 [file aging-v9-e84956-s002.pdf]

Multimedia Appendix 2. Correlations between behavioral attitude dimensions and the overall scale score

Behavioral attitude dimension Correlation coefficient (r)	Behavioral attitude dimension Correlation coefficient (r)
F1 Alleviating burden	0.762
F2 Enhancing convenience	0.701
F3 Service acceptance	0.558
F4 Digital trust	0.414
F5 Funding sources	0.489
