# Supplementary material for: Attitudes Toward Digital Meal Assistance Services Among Older Adults in China: Cross-Sectional Survey
Source: JMIR Aging. 2026 Mar 30;9:e84956. doi: 10.2196/84956 (PMC13035262; doi:10.2196/84956)
Supplement: Checklist 1 [file aging-v9-e84956-s003.pdf]

Checklist 1 CHERRIES checklist

This checklist is completed according to the CHERRIES (Checklist for Reporting Results of Internet E-Surveys) guideline for the online component of the mixed-mode survey (Wenjuanxing). Locations refer to the revised manuscript section titles used in the Methods section.

Design

Item: Open vs closed survey

Response: The online component was an open survey administered via Wenjuanxing. Access was provided through a web link distributed primarily by community staff, with additional responses collected via Wenjuanxing's questionnaire mutual-assistance service. No login was required.

Location in manuscript (Methods): Online survey procedures and quality control

Item: Contact mode

Response: Participants accessed the questionnaire through a web link on Wenjuanxing.

Location in manuscript (Methods): Online survey procedures and quality control

IRB (Institutional Review Board) Approval and Informed Consent

Item: Ethics approval

Response: The study involved minimal-risk anonymous survey research. According to institutional policy and local practice, formal IRB review was not required, and an IRB was not available at the authors' institution for this type of study. The study was conducted in accordance with the Declaration of Helsinki.

Location in manuscript (Methods): Ethics Approval and Informed Consent

Item: Informed consent

Response: For the online survey, consent was implied by submission of the completed questionnaire. An information page described the study purpose, procedures, voluntary participation, and data protection measures.

Location in manuscript (Methods): Ethics Approval and Informed Consent

Item: Data protection

Response: No personally identifiable information was collected. Online responses were exported and stored in password-protected files accessible only to the research team.

Location in manuscript (Methods): Data Privacy and Security

Development and Pretesting

Item: Development and pretesting

Response: Questionnaire items were developed based on prior literature and refined prior to formal data collection.

Location in manuscript (Methods): Measures

Recruitment Process and Description of the Sample

Item: Recruitment process

Response: Survey links were distributed primarily by community staff to older adults living in the study area. A proportion of responses were additionally collected through Wenjuanxing's questionnaire mutual-assistance service.

Location in manuscript (Methods): Online survey procedures and quality control

Item: Incentives
Response: No incentives were provided.
Location in manuscript (Methods): Online survey procedures and quality control
Item: Eligibility criteria
Response: Eligibility was restricted to adults aged ≥60 years. Respondents reporting age <60 were automatically exited from the questionnaire using an automated screening item.
Location in manuscript (Methods): Online survey procedures and quality control
Item: Sample description
Response: The final analytic sample included 405 valid online questionnaires and 614 valid offline questionnaires (N=1,019).
Location in manuscript (Methods): Study Design and Setting

Survey Administration
Item: Survey administration
Response: The online survey was administered via Wenjuanxing.
Location in manuscript (Methods): Online survey procedures and quality control
Item: Mandatory/voluntary participation
Response: Participation was voluntary.
Location in manuscript (Methods): Ethics Approval and Informed Consent
Item: Time/date
Response: The survey was conducted in 2020 over a two-month period.
Location in manuscript (Methods): Study Design and Setting
Item: Adaptive questioning
Response: An automated screening question exited respondents younger than 60 years.
Location in manuscript (Methods): Online survey procedures and quality control
Item: Number of items
Response: The behavioral attitudes scale included 21 initial items, with 19 retained after factor analysis. Additional items assessed intention and use.
Location in manuscript (Methods): Measures; Data Analysis
Item: Completeness check
Response: Data were checked for completeness before analysis; questionnaires with missing key variables or substantial nonresponse were excluded. The final analytic sample contained no missing values.
Location in manuscript (Methods): Data Analysis

Response Rates
Item: Unique visitors/view rate
Response: Not available because the survey link was disseminated through multiple channels and platform access logs (unique visitors/views) were not accessible to the research team.
Location in manuscript (Methods): Online survey procedures and quality control
Item: Participation rate
Response: Not available because the survey link was disseminated through multiple channels and platform access logs were not accessible to the research team.
Location in manuscript (Methods): Online survey procedures and quality control

Item: Completion rate
Response: The final analytic sample included 405 valid online questionnaires and 614 valid offline questionnaires (N=1,019).
Location in manuscript (Methods): Study Design and Setting

Preventing Multiple Entries
Item: IP check
Response: Yes. Submissions were restricted to one response per IP address to prevent multiple entries.
Location in manuscript (Methods): Online survey procedures and quality control
Item: Cookies
Response: Not used / not reported.
Location in manuscript (Methods): Not reported
Item: Log file analysis
Response: Not used / not reported.
Location in manuscript (Methods): Not reported
Item: Registration
Response: Not required.
Location in manuscript (Methods): Not reported

Analysis
Item: Handling of incomplete questionnaires
Response: Questionnaires with missing key variables or substantial nonresponse were excluded. The final analytic sample contained no missing values.
Location in manuscript (Methods): Data Analysis
Item: Statistical correction
Response: Not applicable.
Location in manuscript (Methods): Not applicable
